# Visual processing speed in hemianopia patients secondary to acquired brain injury: a new assessment methodology

**DOI:** 10.1186/s12984-020-0650-5

**Published:** 2020-01-31

**Authors:** Laura Mena-Garcia, Miguel J. Maldonado-Lopez, Itziar Fernandez, Maria B. Coco-Martin, Jaime Finat-Saez, Jose L. Martinez-Jimenez, Jose C. Pastor-Jimeno, Juan F. Arenillas

**Affiliations:** 1 Universidad de Valladolid, Valladolid, Spain; 2 Instituto Universitario de Oftalmobiología Aplicada (IOBA), Eye Institute, Universidad de Valladolid, Valladolid, Spain; 3 CIBER BBN, National Institute of Health Carlos III, Madrid, Spain; 4 Department of Neurology, Hospital Clínico Universitario de Valladolid, Valladolid, Spain; 5 ASPAYM-Castilla y Leon Foundation, Research Centre for Physical Disabilities, Valladolid, Spain; 6 Department of Ophthalmology, Hospital Clínico Universitario de Valladolid, Valladolid, Spain

**Keywords:** Visual processing speed, Hemianopia, Visual ability, Homonymous visual field defects, Acquired brain injury, Eye-hand coordination, Saccadic eye movements, Visual assessment, Neurovisual rehabilitation

## Abstract

**Background:**

There is a clinical need to identify diagnostic parameters that objectively quantify and monitor the effective visual ability of patients with homonymous visual field defects (HVFDs). Visual processing speed (VPS) is an objective measure of visual ability. It is the reaction time (RT) needed to correctly search and/or reach for a visual stimulus. VPS depends on six main brain processing systems: auditory-cognitive, attentional, working memory, visuocognitive, visuomotor, and executive. We designed a new assessment methodology capable of activating these six systems and measuring RTs to determine the VPS of patients with HVFDs.

**Methods:**

New software was designed for assessing subject visual stimulus search and reach times (S-RT and R-RT respectively), measured in seconds. Thirty-two different everyday visual stimuli were divided in four complexity groups that were presented along 8 radial visual field positions at three different eccentricities (10^o^, 20^o^, and 30^o^). Thus, for each HVFD and control subject, 96 S- and R-RT measures related to VPS were registered. Three additional variables were measured to gather objective data on the validity of the test: eye-hand coordination mistakes (ehcM), eye-hand coordination accuracy (ehcA), and degrees of head movement (dHM, measured by a head-tracker system). HVFD patients and healthy controls (30 each) matched by age and gender were included. Each subject was assessed in a single visit. VPS measurements for HFVD patients and control subjects were compared for the complete test, for each stimulus complexity group, and for each eccentricity.

**Results:**

VPS was significantly slower (*p* < 0.0001) in the HVFD group for the complete test, each stimulus complexity group, and each eccentricity. For the complete test, the VPS of the HVFD patients was 73.0% slower than controls. They also had 335.6% more ehcMs, 41.3% worse ehcA, and 189.0% more dHMs than the controls.

**Conclusions:**

Measurement of VPS by this new assessment methodology could be an effective tool for objectively quantifying the visual ability of HVFD patients. Future research should evaluate the effectiveness of this novel method for measuring the impact that any specific neurovisual rehabilitation program has for these patients.

## Background

Vision is the dominant sensory function in humans because visual search and reach tasks are crucial to efficient performance of the main activities of daily life [1, 2]. The term visual processing speed (VPS), an important variable of visual sensory function, is the *amount of time* needed to make a *correct interaction* with a visual stimulus [3, 4]. The term *correct interaction* is the effective realization of a complete executive action of visual search and reach [5], e.g., visualizing a glass of water placed on a table and then grasping it by precise eye-hand coordination (EHC). Accordingly, the VPS variable defines the global reaction time (RT) that is composed of two additive RT sub-variables: search reaction time (S-RT) and reach reaction time (R-RT) [6–8]. Furthermore, VPS is mainly interdependent on intrinsic visual cognitive processing mechanisms, the complexity of the determined stimulus to be recognized (defined principally in terms of size, contrast, semantic content, and number of traces or interior angles [9, 10]), the number of distractor stimuli surrounding it, and the distance from the point of fixation to the particular stimulus that the person is tasked to identify (eccentricity) [4, 11–13]. Thus, VPS is a quantifiable parameter that objectively reflects a subject’s global visual ability.

Recent findings in the field of visual psychophysics show that having adequate VPS is necessary and dependent upon the proper functioning of six main brain-processing systems: auditory-cognitive, attentional, working-memory, visuocognitive, visuomotor, and executive [14–18]. Consequently, an acquired brain injury (ABI) that affects any of these cerebral processing systems could decrease the VPS.

ABI is one of the most important and disabling public health problems of our era due to the high incidence and prevalence [19]. Following an ABI, between 30 and 85% of patients will experience some type of visual dysfunction [20, 21], especially homonymous visual field defects (HVFDs) secondary to lesions involving the visual afferent pathways posterior to the chiasm [22]. Eye tracking technology has shown that HVFDs prevent patients from having the appropriate control of their oculomotor systems [23–26]. This is especially apparent in the saccadic system, because it is interdependent with the covert attention mechanisms associated with peripheral vision [27, 28]. Thus, patients with HVFDs tend to perform search tasks using unconscious compensatory head movements [25, 29, 30] and employ longer total search times, more frequent fixations, and shorter saccades than normal controls [23, 31–37]. Therefore, these patients experience a significant reduction in their quality of life and functional independence. They complain that the time they have to invest in carrying out their daily activities is much greater than before suffering from HVFDs [33, 38–40]. In this regard, in recent years the scientific community has joined efforts to develop increasingly effective neurovisual rehabilitation training programs (NVRTPs) for these patients [41]. Different forms of NVRTPs have been developed, including compensatory NVRTP (C-NVRTP), restitution NVRTP (R-NVRTP), and substitution NVRTP (S-NVRTP) [41–44].

Currently, C-NVRTP is the most accepted therapy because it has greater clinical effectiveness compared to R- and S-NVRTP [41–44]**.** C-NVRTP is based on the theories of brain neuroplasticity, and the main objective is to improve the quality of eye movements in ABI patients. The treatments focus mainly on increasing the amplitude of the saccades and decreasing the number of head compensatory movements and eye fixations that the patients spontaneously and unconsciously tend to perform through their blind visual field [41, 45]. Thus, all C-NVRTPs have a common methodological premise that exposes the patient to searching tasks that must be performed as quickly as possible by moving their eyes and without moving their heads [43, 46–48]. However, no standardized intervention protocol has been developed for C-NVRTPs [41]. Therefore, it is essential that clinicians and researchers in the field of visual sciences develop objective visual ability evaluation methods that will allow them to determine the effectiveness of existing and newly developed NVRTPs [33].

In clinical practice, visual assessment methods, e.g., computerized visual field testing systems, color and contrast sensibility tests, and quality of life questionnaires or scales, are insufficient to determine the real life visual ability of ABI patients [4, 22, 38]. This is because these visual assessment methods tend to minimize distractions and stimulus complexity, and more importantly, they do not supply results in terms of S-RTs or R-RTs. In research studies, some authors have employed particular visual performance tests to obtain objective results on the impact that specific NVRTPs could have on patient visual ability. These tests are specific computerized methods and/or instruments, e.g., Saccadic fixators, VISIOcoach®, Dynavision®, NVT®, etc., with simple executive action evaluation protocols [43, 46–55]. The tests are designed to quantify changes in RTs when searching and finding luminous stimuli or low complexity stimuli, e.g., like letters or numbers, located in different positions of the visual field. However, according to visual processing theories, simple executive actions used to respond to lights or low complexity stimuli may be insufficient to assess the daily life VPS of patients with HVFDs or even of healthy subjects [10, 56–58]. This is because daily life activities are usually seen as complex executive actions [59] for which correct performance requires the joint activation of the six main cerebral processing systems involved in VPS [14–18]. Consequently, the current computerized evaluation protocols could be overestimating the real visual ability of these patients at the beginning and/or at the end of a specific NVRTP.

Thus, the main purpose of this work was to develop a new assessment methodology that overcomes the limitations of the current methods for evaluating VPS. Therefore, our new method was designed to objectively evaluate the VPS that patients with HVFDs and healthy controls have in their daily life. Accordingly, the evaluation protocol uses RT measurements that assess activation of the six main brain processing systems that determine VPS. Importantly, our newly designed methods provide results that are simple to interpret and require only non-expensive materials.

## Methods

### New assessment methodology: scientific basis, evaluation protocol, and materials

**Fig. 1:**
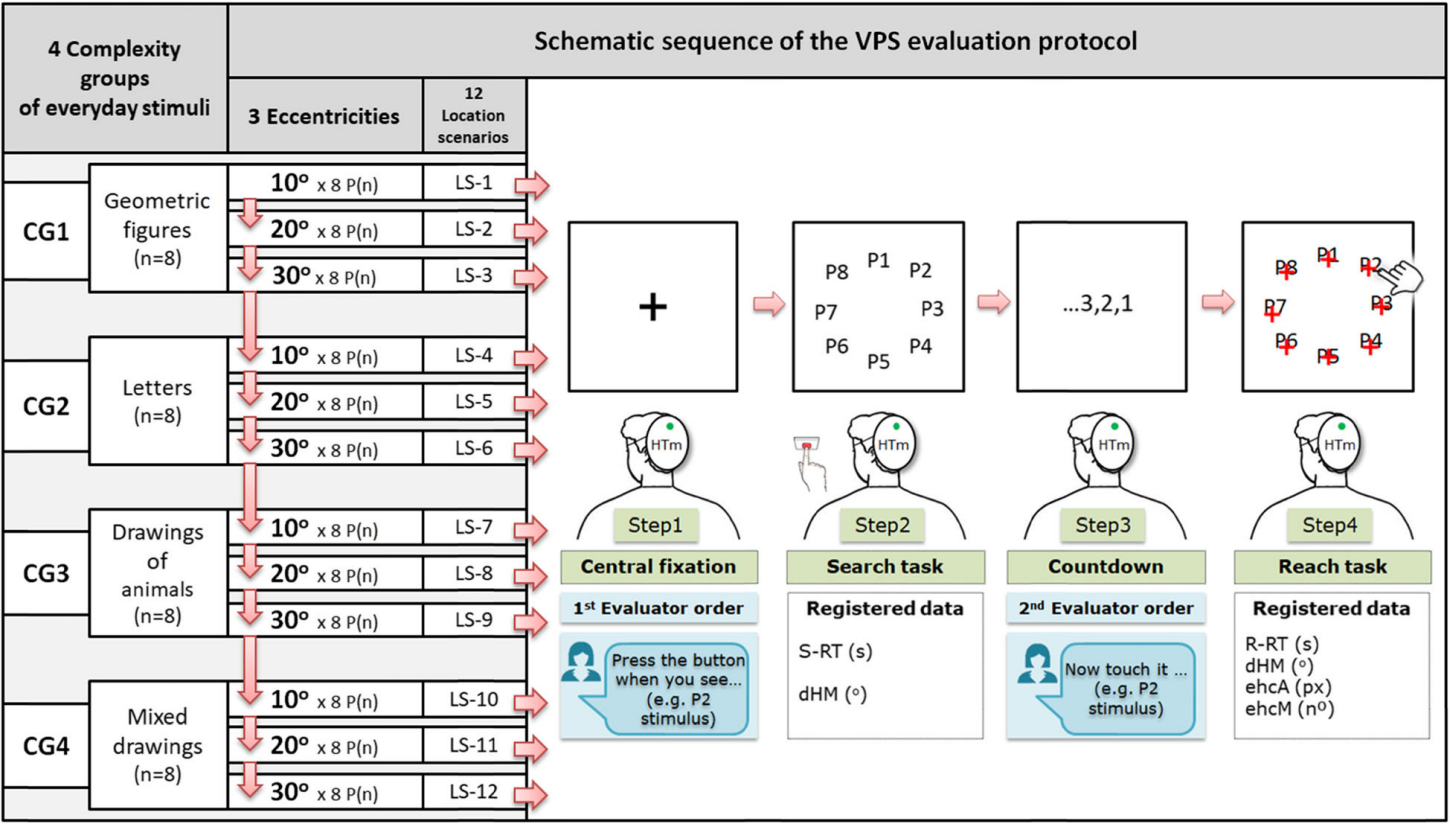
New software was designed and programmed with 12 different sequential location scenarios [LS-(n)] (red arrows). Each LS consisted of 8 stimuli belonging to a specific complexity group [CG(n)]], which were positioned at specific eccentricity (10^o^, 20^o^ or 30^o^) along 8 specific radial positions [P(n)]. Each stimulus position was fixed at each LS-n, but it changed randomly between scenarios. A total of 96 objective measurements were recorded for each one of the six different variables registered during the 4 steps that constituted the new visual processing speed (VPS) evaluation protocol: search reaction time (S-RT; at Step 2), degrees of absolute head movements [dHM; at Step 2 and Step 4 by means of a head-tracker mask (HTm)], reach reaction time (R-RT; at Step 4), eye-hand coordination accuracy (ehcA; at Step 4), and eye-hand coordination mistakes (ehcM; at Step 4)

According to VPS theories [3, 4, 11–18], our new system simulated exposure of the subjects to a controlled situation of a daily search and reach of specific complexity. The visual stimulus was positioned in a specific visuospatial location among a set of distracting stimuli. Furthermore, in accordance with the methodological premises of C-NVRTPs, the subjects should perform the search and reach tasks in the shortest possible time and without moving their heads. Thus, the new software was specifically designed and programmed (Fig. 1) for assessing the subject’s RT in seconds (s) while searching and reaching for 32 different everyday stimuli. The stimuli were presented at 3 different eccentricities (10^o^, 20^o^, and 30^o^) along 8 radial positions (P) of the visual field: P1 at 0^o^ meridian (M), P2 at 45^o^M, P3 at 90^o^M, P4 at 135^o^M, P5 at 180^o^M, P6 at 225^o^M, P7 at 270^o^M, and P8 at 315^o^M. All 32 stimuli were of equal size (2.5 × 2.5 cm or 295.3 × 295.3 pixels) and contrast (99% of white on black). The stimuli were grouped into four complexity groups (CGs) based on two main aspects: (1) the number of traces or interior angles of each stimulus and (2) if they belonged to the same or different semantic families within them. Examples of same semantic family groups in Fig. 1 included CG1 (*n* = 8 geometric figures), CG2 (n = 8 letters), and CG3 (n = 8 drawings of animals). The example containing different semantic family groups in Fig. 1 was CG4 (n = 8 mixed drawings).

Previous studies have shown that humans with high quality mechanisms for inhibiting return and working memory have faster RTs while searching for the stimuli [18, 30, 60, 61]. Thus, our new assessment method simulated exposure of study subjects to daily life situations that required the activation of inhibition of return and short-term memory mechanisms. To achieve this, 12 different sequential location scenarios (LS-[n]; Fig. 1; red arrows) were created. Eight stimuli belonged to specific CGs positioned at specific eccentricity (10^o^, 20^o^, or 30^o^) along the 8 specific radial positions. Each stimulus position was specific at each LS-n, but it changed randomly between scenarios. Additionally, according to a previous study [62] and the fact that 44^o^ is the maximum average amplitude of a saccadic eye movement that a subject can make without moving his head [54], three eccentricity values were chosen to stimulate the visuospatial brain mechanisms inside three specific visual field areas: central (10^o^), peri-central (20^o^), and peripheral (30^0^).

**Fig. 2:**
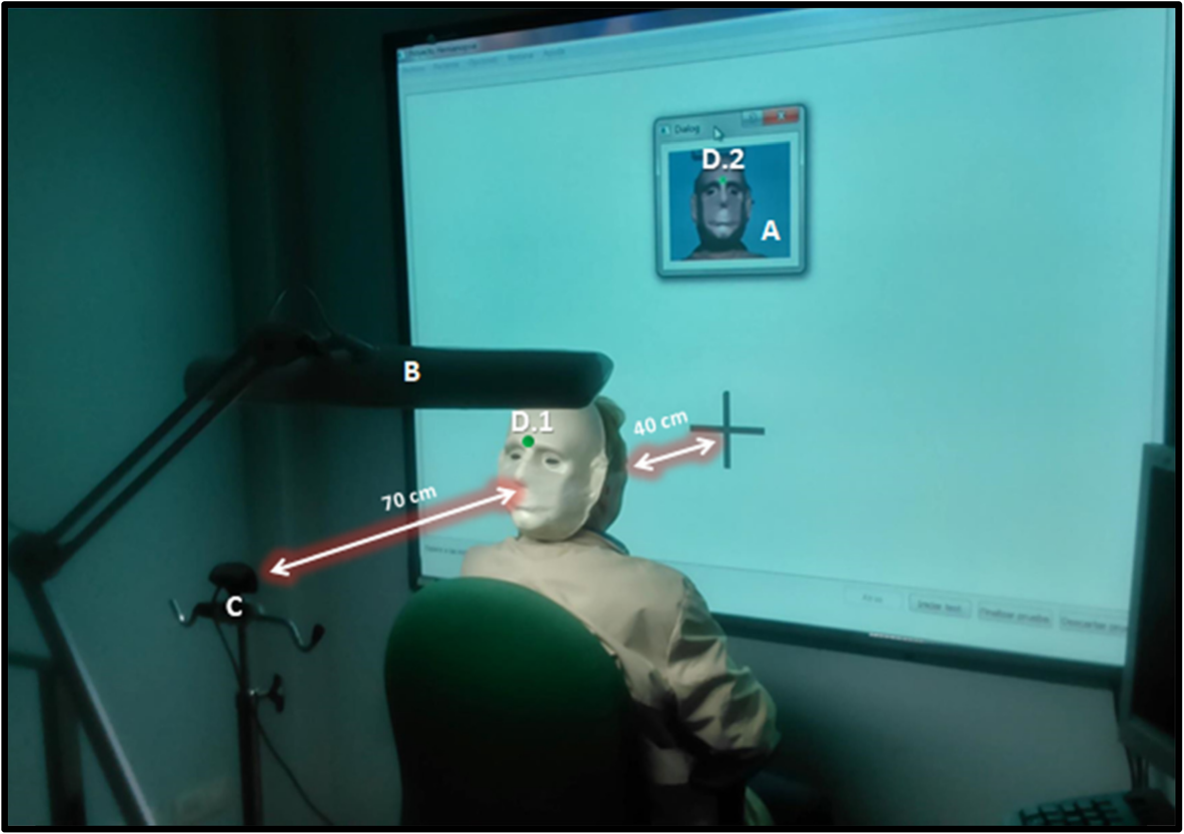
Head Tracker System incorporated in the new software to measure the number of degrees of absolute head movements (dHM) performed by the study subjects, along the coordinate axes “X” and “Y”, while they performed the test. It consisted of specific software capable of detecting human faces (**a**), a fluorescent light (**b**), and a web camera (**c**) that registered the specific movement of a green point placed on a human mask positioned on the back of the subject’s head and neck (**d**.1 and **d**.2). The subject had to remain seated in front of the digital resistive-touch whiteboard at a distance of 40 cm (15.7 in.) and at 70 cm (27.5 in.) from the webcam

To obtain objective data on the validity of the test, two additional variables were included: eye-hand coordination mistakes (ehcM) and eye-hand coordination accuracy (ehcA, measured in pixels). ehcA values were recorded only when patients touched the digital board within an area surrounding the correct stimulus (30 × 30 pixels). When touching outside this area, the time kept running, and the system recorded an ehcM. Additionally, a head tracker system was incorporated (Figs. 1 and 2) to objectively record how the subjects performed the test according to the requirement of not moving their heads. The head tracker system determined the number of degrees of head movements (dHM) performed along the coordinate axes “X” and “Y” during the assessment.

The VPS evaluation protocol was divided into 4 main steps (Fig. 1).

- Step 1: The subjects were asked to remain seated and motionless in front of the digital board at a distance of 40 cm (15.7 in.), with their gaze specific and centered with respect to a black cross (60 × 60 pixels). Simultaneously, the evaluator informed them verbally of the specific stimulus that they had to recognize during Step 2. Accordingly, the correct performance of this step required activation of the alert-concentration system and the auditory-cognitive system [63].
- Step 2: Eight different stimuli appeared in 8 different positions with a specific eccentricity (10^o^, 20^o^, or 30^o^). In addition, the system registered values related to the possible unintentional dHMs while they were searching for the specific stimulus announced during Step 1. Once the subjects visualized the correct stimulus among the 7 distractors and pressed the “enter button”, the assessment system automatically registered one S-RT and all stimuli disappeared. Therefore, correct performance of this step required activation of the alert-concentration system and attentional networks linked to visual-cognitive, visuomotor, and executive processing [14, 64–66].
- Step 3: A 5-s countdown was displayed, starting after pressing the button during Step 2 and before Step 4 began. At this step, it was necessary to keep active the brain-processing mechanisms related to the attentional networks and short-term memory [18, 63, 65]. The subject had to remain motionless, vigilant, and focused both on remembering the stimulus and its location and on mentally planning the act of EHC that he had to carry out in the following step.
- Step 4: The 8 stimuli appeared once again in the same position and eccentricity as in Step 2, but with a red cross (12.8 × 12.8 pixels) in the center of each one. The subjects had to touch the whiteboard on the red cross of the stimulus previously visualized in Step 2 with a finger from their dominant hand or their non-affected hand (if the ABI had affected the mobility of their dominant hand). This task was to be accomplished in the shortest possible time while not moving their head. Data for four variables were collected after this step: (1) R-RT, including the period of time after the 8 stimuli appeared until the study subject touched the correct one; (2) ehcA related to the distance in pixels from the center of the stimulus of the touch location on the tactile whiteboard; (3) dHM made by the subject since the beginning of Step 4 until a successful attempt was made; and (4) the number of ehcMs made by the subject until the right stimulus was touched. Hence, the correct performance of Step 4 required activation of the alert-concentration system, short-term memory, inhibition of return mechanisms [18, 30, 60, 61], attention networks, and the mental constructs linked to visuocognitive, visuomotor, and executive processing [14, 64–66].

Thus, a total of 96 objective measurements were recorded for each one of the six different variables that were registered during the 4 steps (Fig. 1).

Four main pieces of programed software were used to develop the new assessment methodology: C ++®, Open CV®, SQLite®, and Qt®. The hardware consisted of 7 main instruments (Fig. 2): (1) a digital resistive-touch whiteboard (Multiclass Board Touch® 78“); (2) an ultra-short distance projector (Epson EB-470®); (3) a central processing unit (Visa Hermes® CPU Intel Pentium Dual Core with 2GHz and 1 GB of RAM); (4) a high-performance webcam [Trust USB 2.0 eLight Full HD 1080P®, with 2 megapixel (1920 x 1080), high-definition video resolution, and image capture resolution of up to 8.3 megapixels (3840 x 2160)]; (5) a head-tracker mask; (6) a light emitting diode lamp, with adjustable arm and head, of 42 W and a luminosity of 3,760 Lux at 30 cm; and (7) a keyboard (Logitech ®), for which the “enter button” acted as a push button.

### Subjects and study groups

This prospective, interventional study was approved by the Clinical Research Ethics Committee of the Health Area of Valladolid-CEIC-VA-ESTE-HCUV, code PI-17-849 CINV 13–46. All subjects signed an informed consent document before participating in the study.

Thirty ABI patients and 30 healthy control subjects were recruited from four clinical centers: IOBA-Eye Institute of the University of Valladolid, Hospital Clinico Universitario of Valladolid, Hospital Universitario Rio Hortega of Valladolid, and ICTIA (an association of ABI patients of Valladolid, Spain). Each ABI patient was matched in age and gender to their respective control subject. The main common inclusion criteria for both groups were ages between 25 and 85 years old; neuro-ophthalmological, neurological, and radiological clinical stability (≥ 3 months after the ABI); with far binocular visual acuity ≥0.1 LogMAR (ETDRS Chart©); without visual hemineglect (clock-drawing test [67] and line bisection test [68]) visual agnosia (Poppelreuter-Ghent’s© test [69, 70]) or cognitive deficit (mini-mental state examination (MMSE) [71]); with sufficient residual hearing and hand-arm ability to be able to complete the new assessment system without special assistance. All enrolled ABI patients had HVFDs, including homonymous hemianopia (HH) or quadrantanopia (HQ), but they had no previous NVRTP. Control healthy subjects had normal monocular perimetry using the SITA-central standard 30–2 test of the Humphrey® perimeter. The main descriptive parameters of ABI patients are shown in Table 1. Both study groups were assessed in a single visit.

**Table 1:** Acquired brain injury group demographics

Mean ABI duration, m (SD)	24.7	(30.6)
Mean age, years (SD)	57.7	(16.3)
Gender, n (%)		
Male	18	(60)
Female	12	(40)
HVFD side, n (%)		
Left	14	(46.7)
Right	16	(53.3)
Defect type, n (%)		
Hemianopia	21	(70)
Quadrantanopia	9	(30)
Etiology, n (%)		
Ischemic stroke	13	(46.4)
Hemorrhage	3	(10.8)
Tumor	6	(21.4)
Intracranial aneurysm	6	(21.4)
Hand motor disability, n (%)		
Dominant hand	7	(23.3)
Non-dominant hand	7	(23.3)
Without disability	16	(53.3)

*ABI* acquired brain injury, *m* months, *SD* standard deviation, *n* number, *HVFD* homonymous visual field defect

### Statistical analysis

The statistical analysis was performed using the statistical package R, version 3.5.1 (R Core Team, 2018), and the significant value was established for *p* ≤ 0.05.

To carry out the statistical analysis, the reaction times of Step 2 and Step 4 (S-RT and R-RT respectively) were added for each of the 32 existing stimuli in each of the 3 evaluated eccentricities, obtaining a total of 96 different values for the VPS for each subject.

**Fig. 3:**
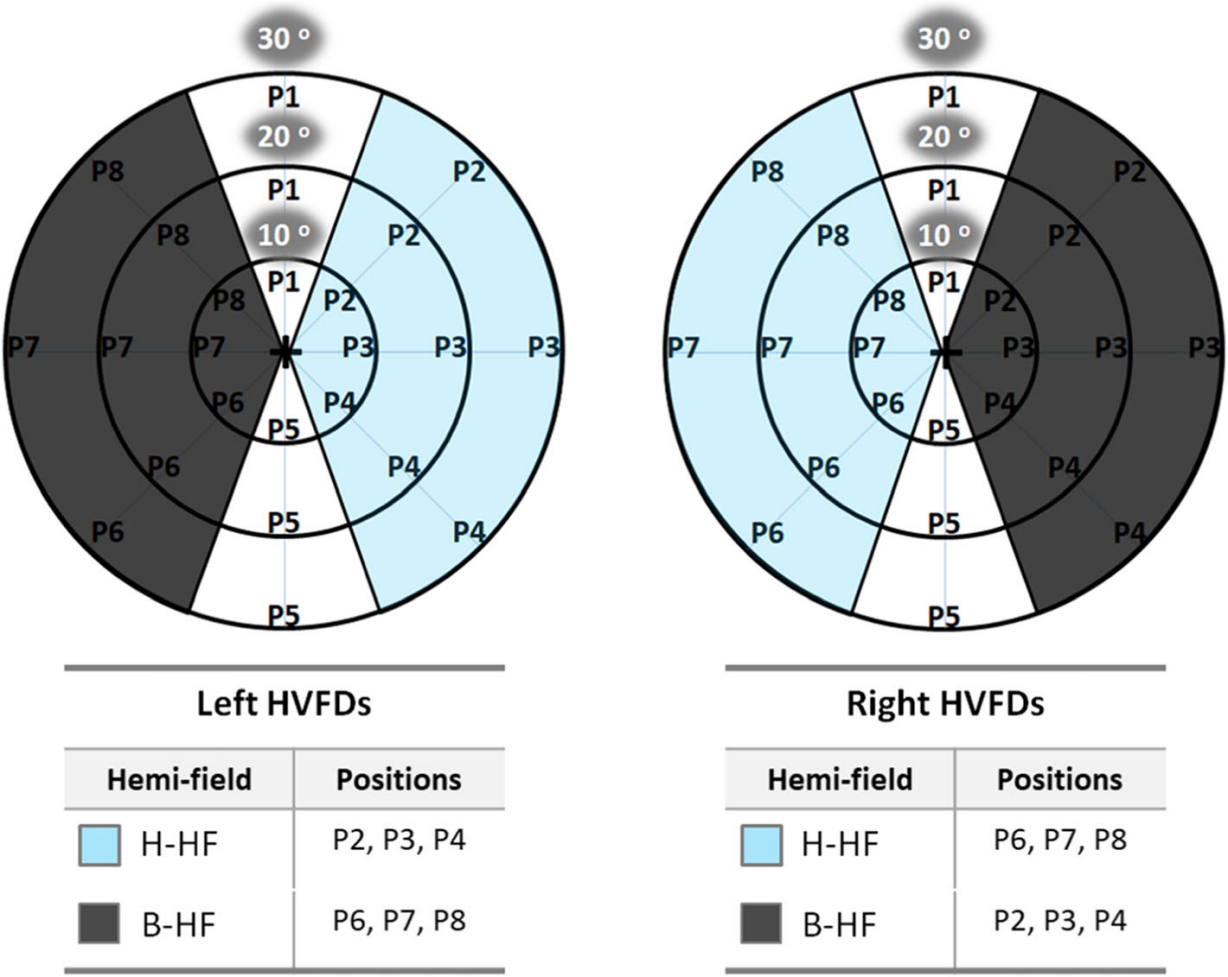
Schematic diagrams representing each of the 8 radial positions in which study subjects had to search and reach 32 visual stimuli from 3 eccentricities (10^0^, 20^0^, and 30^0^). To statistically compare the visual processing speed (VPS) between the visual hemifields in the acquired brain injury (ABI) group, positions P2, P3, and P4 were considered as a healthy hemifield (H-HF, in blue) for the left homonymous visual field defects (HVFDs) and blind hemifield (B-HF, in gray) for right HVFDs. On the other hand, positions P6, P7 and P8 were considered as H-HF for right HVFDs and blind B-HF for left HVFDs. Positions P1 and P5 were not considered for this statistical analysis because they involved both HF

The data analysis for the VPS variable was done by grouping the 96 total values in six different ways (Figs. 1 and 3): (1) total VPS in the complete test, (2) VPS for each of the four CG stimuli, (3) two-by-two comparisons among the four CG stimuli, (4) VPS for each of the three eccentricities, (5) two-by-two comparisons among the three eccentricities, and (6) VPS for the blind hemifield (B-HF) and the healthy hemifield (H-HF). The validity of the VPS data was tested by analyzing the values in the complete test based on the number of ehcMs, ehcAs, and dHMs.

Student’s t-test for paired samples was used to compare both study groups as two dependent samples. When the normality of the distribution of the differences for the matched pairs was not valid, the non-parametric alternative, the Wilcoxon signed-rank test, was used. Normality assumptions were checked by the Shapiro-Wilk test. The difference between the two groups was quantified using the mean of the differences and a 95% confidence interval (CI) for that mean. The relative percentage of change per subject was calculated as $\mathit{\mathsf{Percentage}\ \mathsf{of}\ \mathsf{Change}}=\left(\left({\mathit{\mathsf{Value}}}_{\mathit{\mathsf{ABI}}}-{\mathit{\mathsf{Value}}}_{\mathit{\mathsf{Control}}}\right)/{\mathit{\mathsf{Value}}}_{\mathit{\mathsf{Control}}}\right)\times \mathit{\mathsf{100}}$*.* The one sample Student’s t-test was used to check the hypothesis for the average change, which was 0.

Repeated measures ANOVA were used to compare intra-subject factors within the ABI and control groups. In addition to the normality hypothesis, in the ANOVA model with repeated measures it was necessary to suppose that the variances of the differences between every two levels of the factor were equal. This was equivalent to assuming that the variance-covariance matrix was circular or spherical. To test this hypothesis, the Mauchly test was used. If the sphericity hypothesis was rejected, an epsilon correction was used, the Greenhouse-Geisser correction. Moreover, a post hoc analysis comparing levels two-by-two was performed. This comparison was made using a Student’s t-test for two related samples, applying the Bonferroni correction to solve the problem of multiple comparisons. If it was not possible to assume the normality hypothesis, the ANOVA test was replaced with repeated measures by the non-parametric alternative, Friedman’s contrast, and pairwise comparisons of levels by the Wilcoxon test.

## Results

The principal results of the study are presented below in terms of study group demographic variables. Further analysis of the VPS variables are shown by grouping the 96 total values in six different ways: (1) total VPS in the complete test, (2) VPS for each of the four CGs of stimuli, (3) two-by-two comparisons among the four CGs of stimuli, (4) VPS for each of the three eccentricities, (5) two-by-two comparisons among the three eccentricities, and (6) VPS for the B-HF and the H-HF in the ABI group. Moreover, ehcM, ehcA, and dHM were analyzed to corroborate the performance of the test.

### ABI and control group demographic variables

The sample consisted of 60 participants among the ABI (*n* = 30) and control (n = 30) groups (Table 1). The groups were matched for age (both groups, 57.7 ± 16.3 years) and gender (60% males and 40% women).

### Total VPS in the complete test

**Fig. 4:**
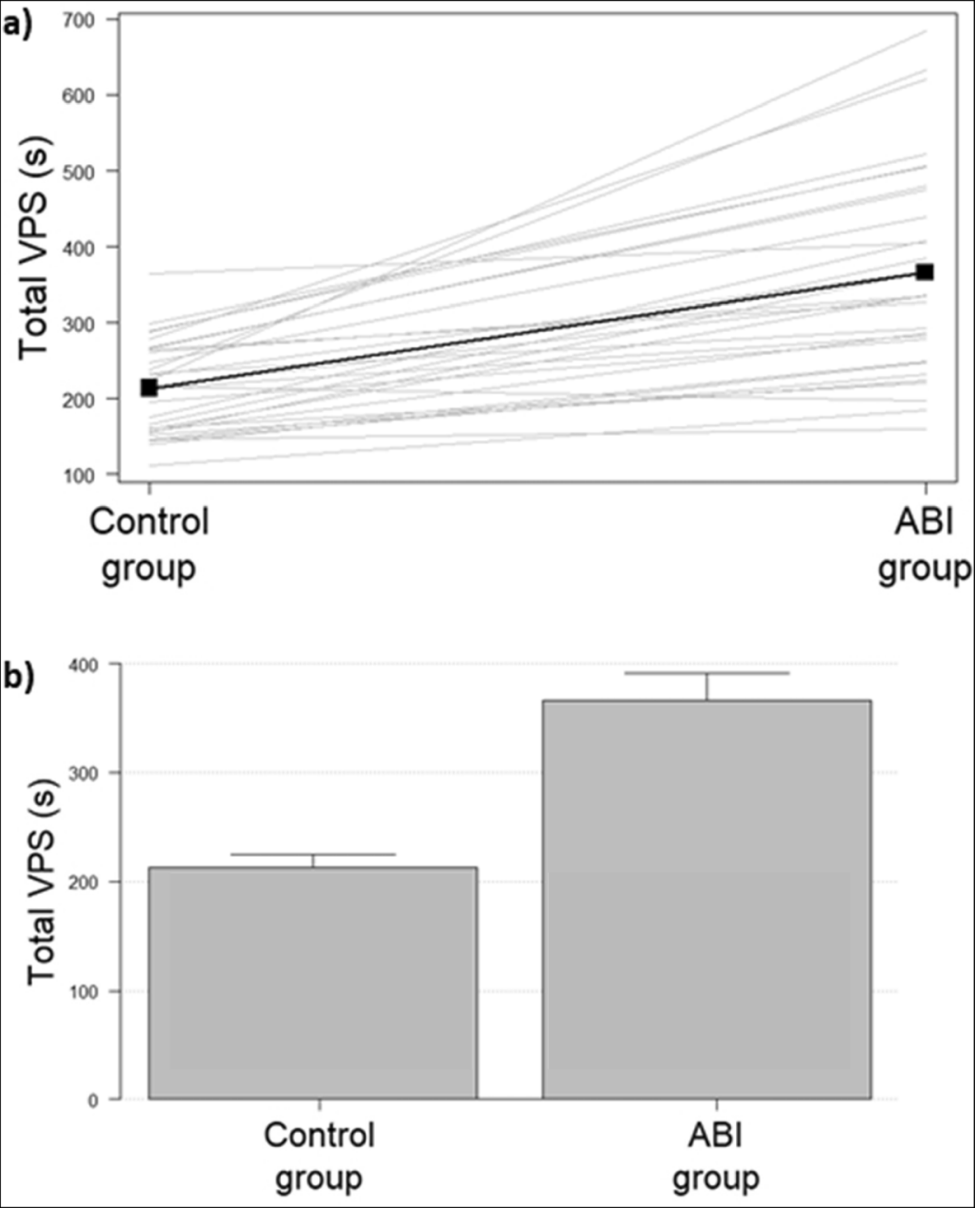
**a** Line segment plots in which each gray line links a pair of mean visual processing speed (VPS) values obtained by each control and an age- and gender-matched acquired brain injury (ABI) patient. Thus each gray line shows the VPS responses (at the left and right ends of each straight line). The slope of each line indicates the difference between the components of each data pair. Bold line, the mean trend. **b** It represents graphically the error bars in the two groups

Total reaction times were longer in the ABI group, 366.0 ± 138.6 s, than in the control group, 213.3 ± 61.2 s (*p* < 0.0001, Fig. 4). The total VPS for the ABI patients was 73.0% slower than the controls (95% CI: 54.8%; 90.9%, *p* < 0.0001).

### VPS for each of the four CGs of stimuli

All VPSs for the ABI group were longer than the comparable measurements in the control group (p < 0.0001, Table 2). For all CGs, the ABI group was slower than the control group: 91.5% for CG1, 62.7% for CG2, 75.1% for CG3, and 63.6% for CG4. On the other hand, both groups showed the following sequence of mean reaction times: CG3 > CG1 > CG4 > CG2 (Table 2).

**Table 2:** Visual processing speed for complexity groups of everyday stimuli

CG of everyday stimuli		Group	Mean VPS	CI 95% Mean		*p*-value	CG comparison	Group	VPS change (%)	
			*(s)*	Lower	Upper				Mean	*p*-value
CG1	Geometric figures	ABI	100.1	83.2	116.8	< 0.0001*	CG2 vs CG1	ABI	(−)23.1	< 0.0001*
								Control	(−)12.2	< 0.0001
		Control	51.6	46.7	56.5		CG3 vs CG1	ABI	18.4	0.0005*
								Control	29.0	< 0.0001
CG2	Letters	ABI	72.2	63.0	81.4	< 0.0001*				
							CG4 vs CG1	ABI	(−)17.4	< 0.0001*
		Control	45.1	40.8	49.3			Control	(−)5.3	0.0259
CG3	Animals drawings	ABI	115.4	96.0	135.0	< 0.0001*	CG3 vs CG2	ABI	59.3	< 0.0001*
								Control	47.1	< 0.0001
		Control	67.4	58.6	76.2					
							CG4 vs CG2	ABI	8.8	0.0028*
CG4	Mixed drawings	ABI	78.2	68.2	88.3	< 0.0001^#^		Control	8.1	0.0030
		Control	49.1	43.5	54.8		CG4 vs CG3	ABI	(−)29.0	< 0.0001*
								Control	(−)25.6	< 0.0001

Table 2 summarizes both study groups mean VPS values (s), its CI 95% and the p-values obtained from comparing both samples for each of the 4 CGs of everyday stimuli. On the other hand, it includes for each study group, the mean VPS % of change values and the *p*-values obtained from each one of the 6 possible two-by-two comparisons among the 4 types of stimuli

*CG* complexity group, *s* seconds, *VPS* visual processing speed, *CI* confidence interval, *ABI* acquired brain injury

* Wilcoxon signed-rank test; ^#^ Paired sample Student’s t-Test

### VPS two-by-two comparisons among the four CGs of stimuli

In both study groups, there were significant differences in the 6 possible two-by-two comparisons among the four CGs of the visual stimuli (*p* ≤ 0.0259, Table 2). Moreover, both ABI and control groups had the highest percentages of change when comparing CG3 vs. CG2. For the ABI group the change was 59.3%, and for the control group it was 47.1%. Curiously, the percentage of change for the control group when comparing CG4 vs. CG2 (8.1%) was very similar to the ABI group (Table 2).

### VPS for each of the three eccentricities

The VPS measured at the 10^o^, 20^o^, and 30^o^ eccentricities were greater for the ABI group than for the control group (*p* < 0.0001 each, Table 3). Thus, the ABI group was 73.0, 67.5, and 79.3% slower than the control group at the 10^o^, 20^o^ and 30^o^ eccentricities respectively. For both groups, the lowest VPS was at 20^o^ (Table 3).

**Table 3:** Visual processing speed for eccentricities

Eccentricity	Group	Mean VPS	CI 95% Mean		*p*-valor	Eccentricity comparison			Group	VPS change (%)	
		(s)	Lower	Upper						Mean	*p*-value
10 ^0^	ABI	124.4	106.4	142.3	< 0.0001^#^	20 ^o^	vs	10 ^o^	ABI	(−)6.0	0.0021*
	Control	72.5	64.3	80.8					Control	(−)3.2	0.0247
20 ^0^	ABI	116.0	99.4	132.6	< 0.0001*	30 ^o^	vs	10 ^o^	ABI	2.0	0.4805*
	Control	69.7	62.3	77.1					Control	(−)1.3	0.4560
30 ^0^	ABI	125.5	107.2	144.0	< 0.0001*	30 ^o^	vs	20 ^o^	ABI	8.5	0.0004*
	Control	71.0	63.4	78.6					Control	2.2	0.1803

Table 3 summarizes both study groups mean VPS values (s), its CI 95% and the p-values obtained from comparing both samples for each of the 3 eccentricities. On the other hand, it includes for each study group, the mean VPS % of change values and the *p*-values obtained from each one of the 3 possible two-by-two comparisons among the 3 eccentricities

*VPS* visual processing speed, *CI* confidence interval, *ABI* acquired brain injury

* Wilcoxon signed-rank test. ^#^ Paired sample Student’s t-Test

### VPS two-by-two comparisons among the three eccentricities

There were significant differences for both study groups when the percent change in mean reaction times at 20^o^ and 10^o^ were compared. The percent decrease in VPS between 20^o^ and 10^o^ for the ABI group, − 6.1%, was about twice the percent decrease of the control group (Table 3). On the other hand, there were no significant differences between the two study groups in the percent change of VPS between 30^o^ and 10^o^. The increase in VPS of the ABI group at 30^o^ compared to 20^o^ was 8.5% (*p* = 0.0004, Table 3), while for the control group it was only 2.2% (*p* = 0.1803).

### VPS for the blind and healthy visual field sides in the ABI group

B- and H-HFs were identified for each ABI (Fig. 3). VPS for the B-HFs were compared with the H-HFs. VPS for the B-HFs, 136.4 ± 56.2 s, were similar to those for the H-HFs, 133.1 ± 51.9 s (*p* = 0.3309).

### Validity of the VPS measurements

#### Number of ehcM

The ehcM for all of the VPS measurements in the ABI group was 0.03 ± 0.20 with errors in the range of 0–3. For the control group, the ehcM was 0.01 ± 0.10 with errors in the range of 0–2. The ABI group had 335.6% more errors (5.1 ± 4.1 failures/subject) than the control group (1.2 ± 1.7 failures/subject, *p* < 0.0001).

#### Accuracy in the eye-hand coordination

The ehcA for all of the VPS measurements in the ABI group was 4.4 ± 4.9 pixels (range, 0–21 pixels). For the control group, the ehcA was 3.4 ± 4.1 pixels (range, 0–17 pixels). Thus, the ABI group was 41.3% more inaccurate (847.0 ± 117.7 pixels/subject) than the control group (652.2 ± 187.0 pixels/subject, *p* < 0.0001).

#### Degrees of absolute head movement performed

For the ABI group, dHM for all of the VPS measurements was 1.0 ± 1.3 degrees (range, 0.1–29.0 degrees). For the control group, the dHM was 0.4 ± 0.7 degrees (0.03–9.4 degrees). Thus, considering all of the dHMs in entire assessment test, which included 96 dHMs in Step 2 and 96 dHMs in Step 4, the degrees of head movement for the ABI group were 189.0% more (94.7 ± 62.8 degrees/subject) than for the control group (42.9 ± 33.3 degrees/subject, *p* = 0.0007).

## Discussion

To the best of our knowledge, this is the first assessment methodology based on VPS theories that has been specifically designed to objectively measure the impact that HVFDs have on patient visual ability. Consequently, our results are only comparable with previous studies in general terms. Previous studies, using subjective quality of life questionnaires, found that the main complaint of these patients is their difficulty of scanning scenes fast enough to have adequate visual cognition of their surroundings [33, 41]. Importantly, for the first time, our new system allows for an objective demonstration of these complaints. In our ABI cohort, consisting of a specific and extensive sample of patients with HVFDs, the VPS was 73% lower than in age and gender matched controls.

Our findings are consistent with other studies, using objective methods such as eye tracking systems, that demonstrated that patients with HVFDs tend to scan visual scenes employing longer total search times, more frequent fixations, and shorter saccades than visually-normal controls [23, 31–37]. In our case, the new system was equipped with a specific head-tracker device that recorded 189.0% more dHMs in the ABI group than in the controls even though both groups were instructed before and repeatedly during the test to keep their heads still. These findings are in accordance with previous studies employing driving simulators in which HVFD patients made more head movements towards their B-HF than matched healthy controls [29, 72]. Additionally, the head-tracker verified that both study groups performed the test correctly because the average dHM values did not exceed 3°, even though stimuli were presented at considerably higher eccentricities, i.e., 20° and 30°.

The dHM data are important because they are related to the significantly slower VPS found in the ABI group. Under the constraint of limited head movement, the ABI patients could only achieve the search and reach functions at specific radial positions and eccentricities through eye movements, mainly saccades and fixations. Additionally, the measurements of ehcM and ehcA incorporated in our system showed that the registered VPS values had high validity. The average ehcM of both groups was less than 0.1 mistake, and the average of ehcA did not exceed 10 pixels (2.6 mm). These data, along with the dHM, indicate that each VPS measurement is suitable and has objective validity.

Our results are consistent with the observation that the human spatial-attention system directs the processing information towards the different regions of the extra-personal space [35, 73]. Moreover, covert attention networks allow subjects to attend to an extra-foveal region of space without a change of fixation, and at the same time, this covert attention is necessary for the correct performance of overt attention networks responsible of the oculomotor system [5, 27, 59, 65]. Thus, the largest proportion of the ehcMs of the ABI group could be strongly associated with an inappropriate self-guided visual exploration of the natural environment. This could possibly be due to (1) spatial-attention and working memory deficits that occur between Step 2 and Step 4 where there is a 5 s countdown, (2) a lack of strategic planning leading to chaotic scan-paths, or (3) a slight impairment of object recognition caused by the underlying brain damage [23, 36, 73–75].

The lack of ehcA in ABI patients with HVFDs has been subjectively reported [28, 40]. Those studies found that stroke survivors had poorer EHC, in terms of slower movement and reduced accuracy, when using their affected hand [76]. However, none of our HVFD patients used his/her affected hand to perform Steps 2 or 4, and only 23% of them had to perform these steps with their non-dominant hand. Consequently, our ehcA results could be related to a lack of peripheral vision. Similar results were found by Kotecha et al. in patients with visual field loss due to glaucoma and who exhibited objective deficits in EHC compared with age-matched, normally sighted controls [77]. Furthermore, our results are consistent with recent EHC studies that reveal the fundamental role of peripheral vision in the correct performance of reach tasks [13, 78–80]. In this context, our quantifiable results are consistent with the needs identified by Rizzo et al. who described EHC as one of the most frequent deficits after ABI and highlighted the importance of objective measurements that can serve as sensitive biomarkers within neurological disease processes [78].

According to visual scene complexity theories [9, 81–83], our results objectively demonstrate the existence of differences in the four CG stimuli in our test procedure. For both the ABI and control groups, the VPS was significantly different among the CGs for the 6 possible two-by-two comparisons. However, three critical comparisons provide unique insight regarding the visual processing systems of control subjects and ABI patients. First, for both the ABI and control groups, the VPS decreased in all four CGs in the same order, i.e., CG3 > CG1 > CG4 > CG2. Second, in both groups, the two-by-two CG comparisons with the highest percentage change occurred when the VPS of CG3 (stimuli of the same semantic family: animals) was compared to CG2 (stimuli of the same semantic family: letters). Third, in comparing the different CGs for the two groups, the percent change in VPS for CG4 (stimuli of different semantic family: mixed drawings) and CG2 (stimuli of the same semantic family: letters) were essentially equal. Based on these and other observations, our results objectively suggest that visually normal controls and HVFD patients have similar pathways of visual brain-processing mechanisms even though HVFD patients had significantly lower VPS. Thus, our results reconfirm previous findings in perception research that identified stimulus complexity as one of the most important stimulus properties that affect the RT of the search and task performance [10], speed and accuracy of shape recognition [84], discrimination and reach [85–87], and working memory mechanisms [18, 63, 65, 74, 88].

On the other hand, the lowest VPS percentage of change between the ABI and control groups was for CG2 (62.77%) and CG4 (63.62%). This suggests that as the number of traces or interior angles of everyday visual stimuli decrease and there are more differences between them, the differences in terms of VPS between patients with HVFDs and healthy subjects will be increasingly smaller. For instance, CG4 consisted of stimuli with different semantic families and more traces and interior angles than CG2, which belong to the same semantic family (letters). In this regard, no previous studies have reported how the complexity of visual stimuli affects VPS of patients with HVFDs. Therefore, our work lays the foundations for future research that is relevant to the design of effective NVRTPs and clinical evaluation methods for these patients. Several studies regarding specific NVRTPs have been published in recent years [42–46]. These used low complexity stimuli to train and measure the improvements at the end of the training programs. The authors reported significant improvements in patient RTs, although they do not correspond with global significant improvements in their quality of life as assessed by subjective questionnaires.

According to previous studies [28, 89, 90], the statistical analysis of RTs by eccentricity objectively suggests that HVFDs affect the visuospatial-attention brain processing mechanisms of these patients. Our results are consistent with these studies because the VPS of the ABI patients was significantly slower than the controls at all degrees (72.9% at 10^o^, 67.5% at 20^o^, and 79.3% at 30^o^). Furthermore, for both groups, the lowest average VPS was at 20^o^, which is in accordance with two perceptual phenomena: (1) the *crowding phenomenon* occurs when an object becomes more difficult to identify as the surrounding objects become closer to it [91–93], and (2) the *peripheral vision phenomenon* occurs when an object becomes more difficult to identify as the distance from the subject’s fixation point increases [75, 92]. Additionally, three critical comparisons provide unique insight regarding visuospatial-attention brain processing mechanisms of control subjects and HVFDs patients, as described above for the two-by-two CG VPS comparisons. First, both groups had significantly slower RTs at the central visual field (10^0^) than at the peri-central visual field (20^0^). Thus the *crowding phenomenon* could contribute to decreased VPS more than the *peripheral vision phenomenon* for both groups. Moreover, the decreases in VPS for the ABI group were exactly double the control group. These results could objectively demonstrate why patients with HVFDs complain especially about difficulties in performing daily activities that involve a central visual field stimulation (such as reading and writing [24, 94], looking for specific foods on a supermarket shelf [33], cooking [40], etc. Second, both groups did not have significant differences when comparing VPS values at the central visual field (10°) and at the peripheral visual field (30°). These results suggest that at these eccentricities, the search and reach of the different stimuli (*n* = 32) represents a similar visual processing difficulty for the visually normal controls and the HVFD patients. Third, both groups had higher VPS at 20° than at 30°, but the increase was significant only for the ABI group. These results could be related to covert attention deficits described in ABI patients and patients with visual field defects [3, 62, 95]. In this regard, the control group had no visual field defects, and the covert attention mechanisms were presumably preserved in them. This is likely the reason why the change in VPS between 20° and 30° was not significant. However, results reported by others [3, 35, 62, 95] do not provide data that are relevant to our second and third critical eccentricity comparisons. Thus, they did not report any results between a HVFD and control groups in the VPS values at the central (10°) or peripheral (30°) visual fields. They also did not report comparisons of the VPS at 20° and 30° for both groups. Therefore future studies should attempt to corroborate our findings and progressively design more effective NVRTPs and objective evaluation methods of the visual abilities of HVFD patients.

Finally, based on this new evaluation methodology, we verified that in the ABI group there were no significant differences in VPS of the B- and H-HFs. However, the mean RTs were slightly longer in the B-HF. In the future, this assessment methodology could be applied to patients with severe visual neglect [96] and could determine if the differences between hemifields increase. Such findings may provide objective data to assess the possible impact of specific visual neglect NVRTPs. Additionally, these data are in accord with previous studies utilizing eye tracker systems that found no significant differences in amplitude or frequency of saccades between both hemifields in patients with HVFDs due to unilateral occipital lesions [34, 36]. Based on these results, we propose that NVRTPs should retrain both hemifield visual abilities equally.

### Limitations

The current investigation had several limitations. First, we initially considered the incorporation of an eye-tracker system into the protocol of this study. This resource could have provided valuable information about eye fixations, and it could have corroborated that the study subject was really watching the correct stimulus during Step 1 of the protocol. However, we dismissed this idea for several reasons. The principal one was that our new assessment methodology did not seek to objectify the quality of the patients’ eye movements because there are already numerous studies on this topic [23–26]. Moreover, an eye tracker system could not provide data about involuntary head movements that HVFD patients tend to make towards the B-HF during searching and reaching tasks [25, 29, 30]. Despite the fact that head movements data could provide relevant information regarding the global objective impact of a specific C-NVRTP has on a patient’s visual ability. Additionally, the incorporation of an eye tracker system would increase the cost of the new evaluation system and complicate the evaluation and interpretation of the results. This decision was consistent with our fundamental goal to endow the developed system and protocol with specific evaluation rules, e.g., to keep the head still during the test, four differentiated evaluation steps, and three additional variables, i.e., dHM, ehcM, and ehcA, to generate objective data on how the test was being performed. Second, during Step 1 of the protocol, a subject could falsify the measurement of the S-RT by pressing the keyboard button without having really seen the stimulus. However, Step 4 was designed to obtain the best values of each patient’s VPS, even taking into account a false measurement of the S-RT in Step 1. If the patient had not seen or properly identified the objective stimulus during Step 1, later during Step 4, this fact was recorded by the computer application as an ehcM or as a longer value of R-RT. Certainly, upon completion of the test, our specific assessment methodology demonstrated that the ABI group had 335.6% higher ehcM and 41.3% lower ehcA than the controls.

## Conclusions

Problems in visual brain processing mechanisms after ABI are often a clinical challenge both for diagnosis and treatment [33, 41, 78]. To the best of our knowledge, this is the first assessment methodology specifically designed to objectively value the impact that HVFDs have on patient VPS. Additionally, we determined the efficacy of this new methodology based on objective measurements of VPS, ehcM, ehcA, and dHM. We found significant differences between a large sample of patients with HVFDs and healthy subjects matched by age and sex. Future research will evaluate if this assessment methodology is an effective tool to measure the possible improvements that occur in patients with HVFDs after specific NVRTPs.

## Data Availability

The datasets used and/or analyzed during the current study are available from the corresponding author on reasonable request.

## Abbreviations

ABI: Acquired brain injury
B-HF: Blind hemifield
CG: Complexity group
C-NVRTP: Compensatory neuro-visual rehabilitation training programs
dHM: Degrees of head movement
EHC: Eye-hand coordination
ehcA: Eye-hand coordination accuracy
ehcM: Eye-hand coordination mistakes
H-HF: Healthy hemifield
HVFDs: Homonymous visual field defects
LS: Location scenario
NVRTP: Neurovisual rehabilitation training programs
P: radial positions
R-RT: Reach reaction time
RT: Reaction time
S-RT: Search reaction time
VPS: Visual processing speed
